# Differential effects of prenatal alcohol exposure on brain growth reveals early upregulation of cell cycle and apoptosis and delayed downregulation of metabolism in affected offspring

**DOI:** 10.1371/journal.pone.0311683

**Published:** 2024-11-27

**Authors:** Danielle Sambo, Ethan Kinstler, Yuhong Lin, David Goldman

**Affiliations:** 1 Laboratory of Neurogenetics, National Institute on Alcohol Abuse and Alcoholism, National Institutes of Health, Rockville, Maryland, United States of America; 2 Laboratory of Liver Diseases, National Institute on Alcohol Abuse and Alcoholism, National Institutes of Health, Rockville, Maryland, United States of America; Indiana University-Purdue University Indianapolis, UNITED STATES OF AMERICA

## Abstract

Fetal Alcohol Spectrum Disorder (FASD) encompasses the deleterious consequences of Prenatal Alcohol Exposure (PAE), including developmental delay, microcephaly, dysmorphologies, and cognitive and behavioral issues. The dose and timing of alcohol exposure, maternal and environmental factors, and genetics all impact FASD outcomes, but differential susceptibility and resiliency to PAE remains poorly understood. In this study, we examined the differential effects of PAE during early mouse development on brain growth and gene expression. Brains were weighed and collected either 24 hours or five days after treatment. We then performed transcriptomics to determine whether offspring differentially affected by PAE, by brain weight, also differ in gene expression, despite having the same genetic background, alcohol exposure, and maternal factors. We found within litter variation in brain weights after PAE, and classified offspring as having normal, middle, and low-weight brains relative to saline-treated controls. The normal-weight brains showed no significant differences in gene expression, suggesting these offspring were both phenotypically and transcriptionally unaffected by PAE. While both middle- and low-weight brains showed changes in gene expression, the middle-weight brains showed the most robust transcriptome differences. Twenty-four hours after PAE, we saw an upregulation of cell cycle and apoptosis in affected offspring, whereas at roughly a week later, we saw a downregulation of metabolic processes. Overall, these findings highlight variability in response to PAE and demonstrate the molecular processes involved in offspring phenotypically affected by alcohol.

## Introduction

Prenatal alcohol exposure (PAE) is a major, preventable cause of developmental delay, physical dysmorphia, and neurocognitive deficits. Fetal alcohol spectrum disorder (FASD) encompasses the range of teratogenic effects of alcohol, with fetal alcohol syndrome (FAS) being the most severe form of the disorder. Current estimates reveal that 1–5% of the United States population are affected by FASD [1], with underdiagnosis expected due to inconsistency in screening, the presence of co-occurring conditions complicating diagnosis, and stigma associated with self-reporting alcohol use during pregnancy. Microcephaly, physical deformities, intellectually disability, coordination issues, and behavioral and social difficulties are all part of the constellation of FASD symptoms, and additionally, a number of conditions are comorbid with FASD, attention deficit hyperactivity disorder (ADHD) being the most common [2]. The types of outcomes and their severity are influenced by a number of variables, most prominent being the timing, dose, and duration of PAE. Maternal age, gravidity, parity, nutrition, stress, socioeconomic status, and other lifestyle factors also impact the severity of FASD. Less understood, genetic factors play an important role [3]. Remarkably, comparison of monozygotic and dizygotic twins after PAE has shown 100% concordance of FASD diagnosis for monozygotic twins, whereas dizygotic twins showed only 56–63% concordance, supporting some genetic determination of FASD susceptibility [4, 5]. A genetic contribution is also observed in different animal models, most notably the C57BL/6J strain of mice has been shown to be more vulnerable to PAE than DBA/J mice in numerous studies [6–8]. Even between C57BL/6 sub-strains (6J versus 6N), differences in vulnerability underscore how seemingly subtle genetic differences may impact alcohol teratogenicity [9, 10].

Expanding on the variability of FASD, only 1 in 13 (7.7%) children exposed to alcohol *in utero* develop the disorder [11]. In an early study of women with heavy alcohol use reporting during pregnancy, the estimated incident rate of any abnormality was 50%, with only 2.5% meeting the criteria for FAS [12]. In inbred mouse models of FASD, where maternal factors, genetics, and environmental variables can be controlled, varying responses to the same PAE are observed, both between and within litters. It appears not all offspring exposed to alcohol *in utero* develop FASD or readily observable phenotypes. It is important to note, however, that because the effects of PAE are diverse, they are not comprehensively examined in all studies. PAE, especially at high doses, increases the risk of spontaneous abortions and stillbirths; thus, the most highly affected offspring may not survive. Furthermore, some of the effects of PAE are only apparent later in life, for example ADHD-like phenotypes, and studies may not focus on later cognitive or behavioral deficits. Accurate estimates of the rates and concordance of FASD among those with PAE remains complex and difficult to determine.

In this study, we examined the differential effects of PAE in a mouse model of FASD, investigating whether different phenotypic responses to PAE correspond with differences in gene expression and seeking to identify molecular networks perturbed in proximity to exposure versus later, when deficits may have recovered. We measured brain weight to distinguish between offspring more or less affected by PAE, as the developing brain is especially vulnerable to the effects of PAE due to its prolonged developmental time. Furthermore, microcephaly, or a significantly smaller head, is more common in individuals with PAE [13], especially FAS where it occurs in up to 60% of individuals [14] and predicts poorer cognition later in life [15]. Several mechanisms by which PAE affects brain growth have been proposed, including apoptosis, oxidative stress, disruption of neurotrophic factors, and epigenetic modifications [16]. Via transcriptomics, we measured changes in gene expression and molecular processes affected by PAE in affected and non-affected offspring. By phenotypically characterizing mice for transcriptomics, we hope to better resolve molecular changes by decreasing phenotypic variability and thus enhance our understanding of preclinical models of FASD.

## Materials and methods

### Animal use and prenatal alcohol exposure

All animal procedures were approved by the Institutional Animal Care and Use Committee of the National Institute on Alcohol Abuse and Alcoholism Intramural Research Program. All experiments were performed in accordance with relevant guidelines and regulations. C57BL/6J mice purchased from Jackson Laboratory were maintained on a 12 hour:12 hour light:dark cycle with access to food and water *ad libitum*. Mouse were fed the standard NIH-31 chow diet (7017, Envigo). Timed matings were initiated in the evenings by pairing a single male with a single female. Females were separated the next morning (embryonic day 0.5 or E0.5) and checked for a copulation plug. Female weights were monitored, and pregnant dams, as determined by weight gain, were used for prenatal alcohol or saline exposure. On E7.5, pregnant females were treated with either saline or 2.9 g/kg ethanol (EtOH), delivered in 25% v/v 200 proof EtOH in saline, by intraperitoneal injection (i.p.) once daily for 7 days (E7.5 to E13.5). This dose of EtOH is estimated to result in a blood ethanol concentration (BEC) of 150–200 mg/dl [17] and is considered a moderate dose of EtOH. To measure the more intermediate effects of PAE, dams were euthanized on E14.5 by cervical dislocation without anesthesia, and embryos were collected. To measure the more prolonged effects of PAE, dams treated with saline or EtOH were left undisturbed from E14.5 until birth, or postnatal day 0 (P0), when pups were collected.

### Tissue weights and collection

Whole E14.5 embryos, E14.5 brains, and P0 brains were fixed in 10% formalin for 2 hours at 4°C prior to weighing in order to more easily and accurately weigh the tissue. P0 whole body weights were measured in live pups. After fixation, tissue was briefly dried using a Kimwipe^™^ and weighed. For E14.5 embryos, whole brains were collected after weighing embryos and subsequently weighed. After obtaining whole brain weights for both E14.5 and P0, the frontal cortices from both hemispheres were removed and stored at -80°C until further processing.

### RNA extraction, cDNA library preparation, and library sequencing

To extract RNA from fixed tissue, the RecoverAll Total Nucleic Acid Isolation kit (Thermo Fisher Scientific, AM1975) was used per manufacturer’s instructions. Past studies have successful performed RNA sequencing from RNA extracted from fixed, frozen tissue from human cancer tissue as well as mouse and rat brains [18, 19]. For such studies and in this study, RNA quality is assessed for each sample used to ensure adequate RNA integrity. The quality of RNA was assessed both by an Agilent 21000 Bioanalyzer (Agilent RNA 6000 Pico Kit, Agilent Technologies, 5067–1513) and a NanoDrop 2000 Spectrophotometer (Thermo Scientific). AmpliSeq libraries were generated using the Ion AmpliSeq^™^ Library Kit Plus (Thermo Fisher Scientific, 4488990) per the manufacturer’s instructions. Library quality was assessed using the Agilent High Sensitivity DNA Kit (Agilent Technologies, 5067–4626), and library concentrations were quantified using the Ion Library TaqMan^™^ Quantitation Kit (Life Technologies, 4468802), per manufacturer’s instructions. Barcoded AmpliSeq libraries were loaded on the sequencing chips using the Ion 540^™^ Chip Kit (ThermoFisher, A27766) and Ion Chef^™^ Instrument. Libraries were then sequenced on the Ion Torrent S5 Sequencing System via the Ion Torrent 540-OT2 kit (ThermoFisher, USA, A27753). 8 to 9 libraries were loaded per sequencing chip, and 2 sequencing ships were loaded and sequenced at a time. E14.5 samples and P0 samples were sequenced separately, with Control and PAE samples balanced across chips and sequencing runs. An average of 13.7 million counts per sample were sequenced, with at a minimum of 5 million counts per sample. Sequencing data was summed for libraries which required two sequencing runs to obtain more counts.

### Sex determination

Sex was determined in the E14.5 offspring used for transcriptomics by quantitative PCR (qPCR) for the ubiquitously transcribed tetratricopeptide repeat gene on Y chromosome gene *Uty*, which has been shown to be expressed in the developing mouse cortex selectively in males [20]. Sex determination in P0 pups was performed by visualization of the anogenital distance. Sex was verified by checking the expression of *Uty* and *Xist* via transcriptomic data. On average, there was a 3000X difference in gene expression, as measured by counts, between the two genes in an offspring.

### Gas chromatography mass spectrometry (GC/MS)

To determine the amount of ethanol in offspring brains *in utero*, a separate cohort of dams was used for GC/MS measurement of alcohol content. At E14.5, pregnant dams were treated with 2.9 g/kg of EtOH by i.p. injection. After 15, 30, or 60 minutes, the dams were euthanized by cervical dislocation, and embryo brains were collected, noting the position of the embryo in the uterus (left versus right and position from the cervix (1, 2, 3, etc.)). Because few litters had more than 4 embryos per one uterine horn, only the first 4 positions are reported. Brains were then flash frozen at -80°C until further processing. The GC/MS method was modified from work by Jin et al., 2021 [21]. Brain weights were measured and about 20–30 mg of brain tissue was mixed with 5 μmol of d6-ethanol (internal standard for ethanol) before adding 200 μL of 0.6 N perchloric acid into each sample. Samples were homogenized using the Precellys Evolution tissue homogenizer by shaking on the bead mill at 6800 rpm for 30 s, twice, maintaining a temperature of 4°C and with a 30 second pause between cycles. Samples were then centrifuged at 13,000 g for 15 minutes at 4°C. The supernatant was transferred into a 20-mL headspace vial and capped immediately. Headspace vials were then loaded onto the 111-vial tray of a headspace sampler coupled to a GC/MS instrument (Agilent single quadrupole 5775 Mass Spectrometer coupled with 7890 gas chromatography). The concentration of ethanol was calculated by comparing the integrated areas of ethanol peaks on the gas chromatograms with those of internal standards added in each sample.

### Data analysis

Analysis and generation of graphs of brain and body weights as well as GC/MS data were performed via GraphPad Prism. For AmpliSeq data, alignment and gene expression count were performed using Ion Torrent AmpliSeqRNA Plugin v0.5.4.0 (Thermo Fisher) using mm10 genome. AmpliSeq count data was analyzed using DESeq2 1.34.0 [22]. Genes were excluded which did not have a minimum of 10 counts in at least 12 samples. This filtering resulted in 14,375 genes at E14 and 14,271 genes at P0 that were used for downstream analysis. Batch and sex were controlled for in the DESeq2 experimental design. Results from the differential gene expression (DEG) analysis are provided in the Supporting Information (S2 File Differential Gene Expression Results). Gene expression differences were considered significant at a Benjamini-Hochberg false discovery rate (FDR) adjusted p-value threshold of 0.05. PCA plots were generated using ggplot2 [23], and PCA correlations were performed with pcaExplorer [24]. Differentially expressed genes were used for Gene Ontology (GO) analysis using Database for Annotation, Visualization and Integrated Discovery (DAVID) [25]. Biological processes were considered significant with 3 or more genes and a p-value threshold of 0.05.

## Results

### PAE decrease brain and body weights in E14 and P0 offspring

**Fig 1:**
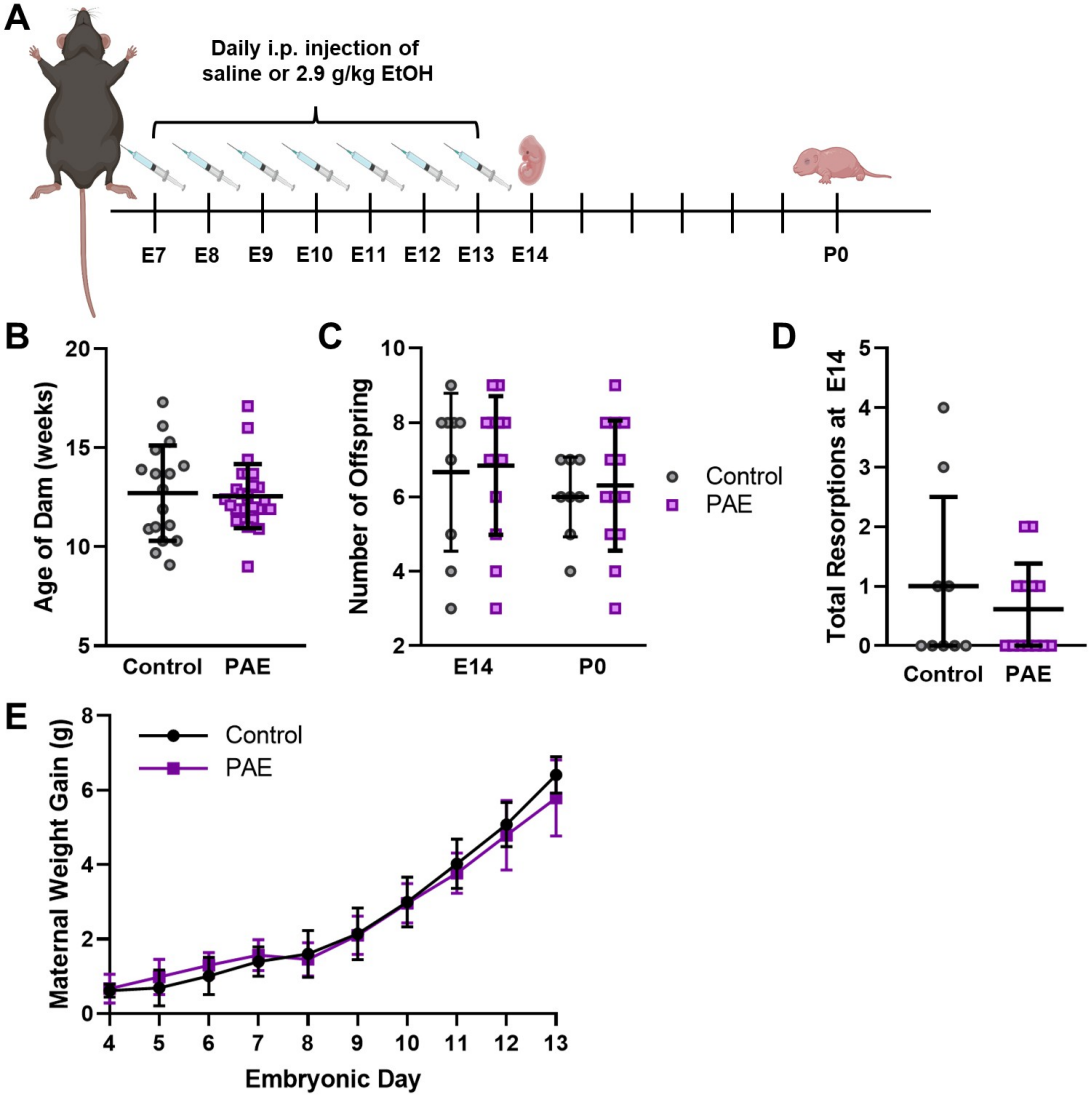
Prenatal alcohol exposure (PAE) paradigm and maternal and offspring characterization. **(A)** Schematic of the PAE paradigm used in this study. Pregnant mice were treated by intraperitoneal (i.p.) injection once daily from embryonic day 7 (E7) to E13 with saline or 2.9 g/kg ethanol (EtOH). Offspring were collected at either E14 or postnatal day 0 (P0). **(B)** Average age of dams for Control (saline treated) or PAE. **(C)** Number of offspring in Control and PAE litters for E14 and P0. **(D)** Total number of resorptions observed at E14 for Control and PAE litters. **(E)** The average maternal weight gain from E4 to E13. Graphics in A were obtained from Biorender.com. (Plots show mean and S.D.).

**Fig 2:**
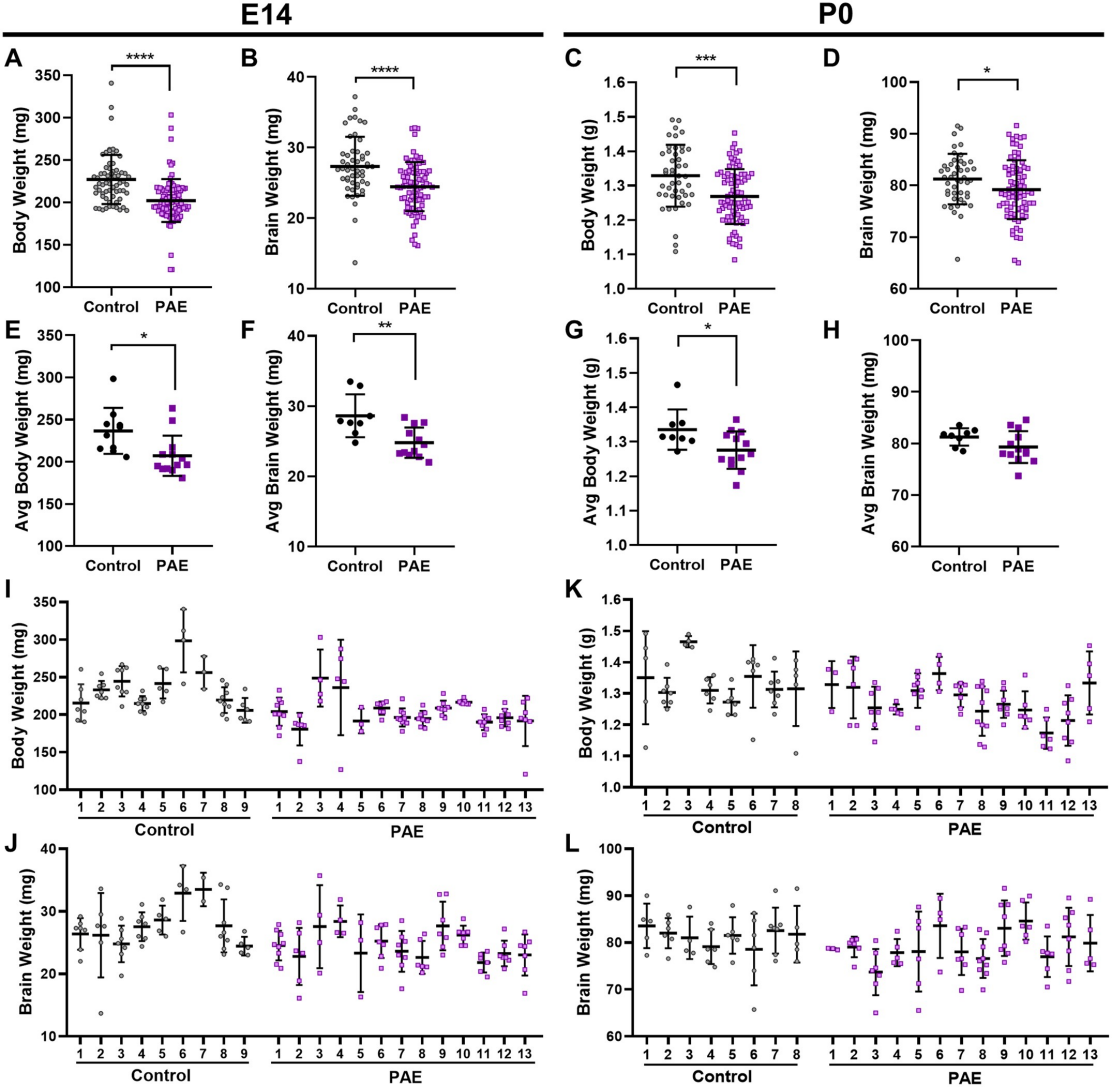
Prenatal alcohol exposure (PAE) decreases brain and body weights at E14 and P0. **(A, B)** Body and brain weights of Control (N = 52) or PAE (N = 84) offspring at E14.5. **(C, D)** Body and weights of control (N = 46) or PAE (N = 80) offspring at P0. **(E, F)** Averaged body and brain weights of Control (N = 9) or PAE (N = 13) offspring by litter at E14. **(G, H)** Averaged body and brain weights of control (N = 8) or PAE (N = 13) offspring by litter at P0. **(I, J)** Offspring body and brain weights by individual litter for control or PAE offspring at E14.5. **(K, L)** Offspring body and brain weights by individual litter for control or PAE offspring at P0. (Scatter plots show mean and S.D.; *: p < 0.05, **: p < 0.01, ***: p < 0.001).

To measure the effects of PAE on brain growth, pregnant C57BL/6J mice were treated with saline or 2.9 g/kg EtOH daily from embryonic day 7 (E7) to E13, representing the start of gastrulation through peak cortical neurogenesis [26]. On E14 or postnatal day 0 (P0), whole body and brain weights of offsprings were measured (Fig 1A). The average number of offspring per dam, resorptions at E14, and maternal weight gain was not different between saline and EtOH (Fig 1B–1E). While this suggests no significant offspring lethality, PAE significantly decreased both body and brain weights at both timepoints (Fig 2A–2D). This effect was observed when comparing average litter weights (Fig 2E–2H), although not statistically significant for P0 brains. While there was inter-litter weight variability, intra-litter variability was also observed (Fig 2I–2L). Weights across all litters were compared by one-way ANOVA with multiple comparisons. At E14, 231 comparisons (117 Control vs PAE, 36 Control vs Control, and 78 PAE vs PAE) revealed 48 significant differences between individual litters for body weight, 33 of which were significant differences between a Control and PAE litter. For brain weight at E14, 13 of 231 comparisons were significant, 12 of which were for Control versus PAE. At P0, of 210 comparisons (104 Control vs PAE, 28 Control vs Control, and 78 PAE vs PAE), 13 were significantly different for body weight, with 10 for Control vs PAE. For brain weight, only one comparison gave significantly different results. These results suggest a greater number of inter-litter differences at E14 compared to P0, with the majority of differences driven by treatment. The average decrease in weights was higher at E14 (12% decrease in body and 10% decrease in brain weight) compared to P0 (4.5% and 2.4% decreases, respectively), indicating some recovery of weight loss may occur after cessation of alcohol.

**Fig 3:**
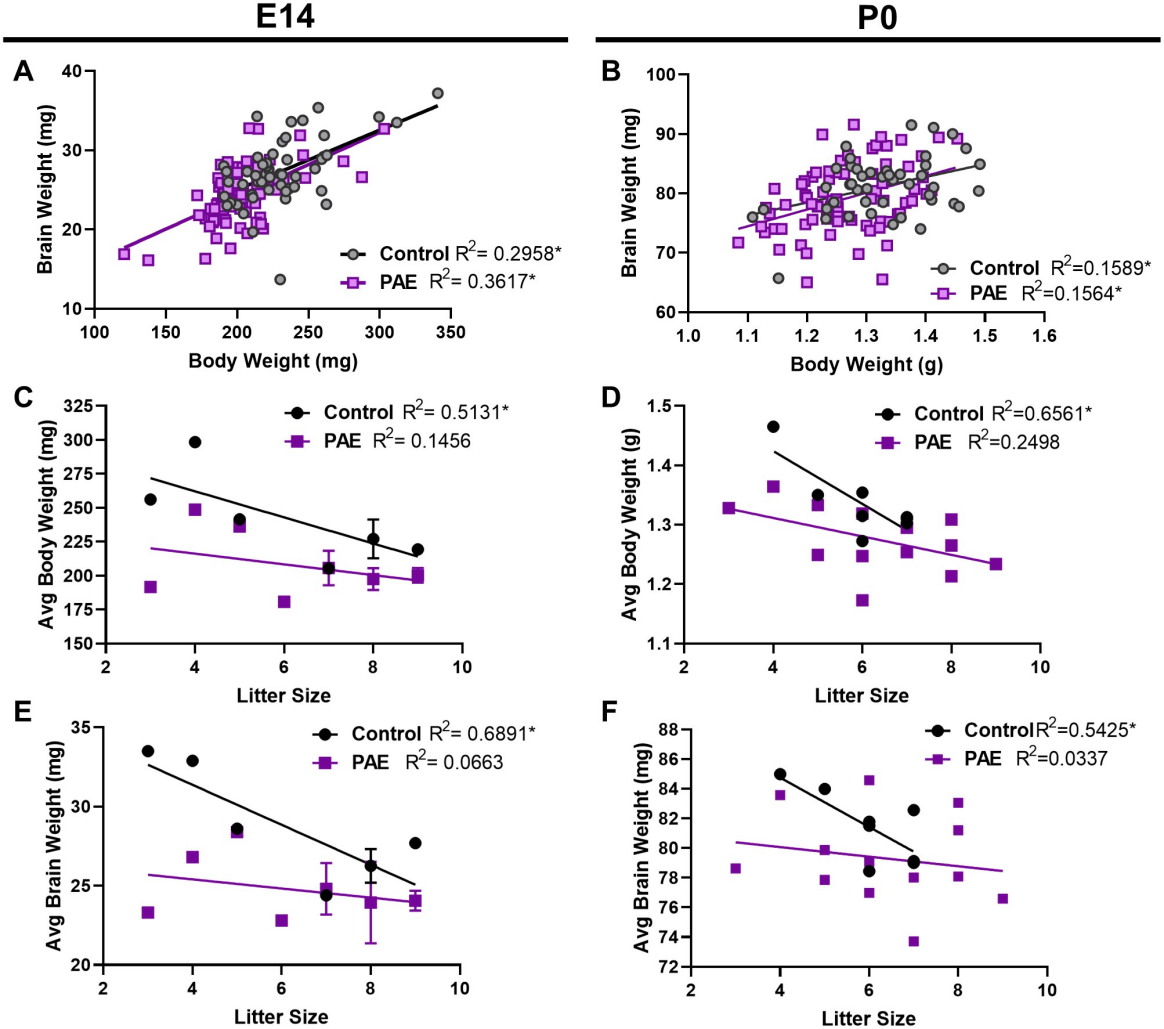
Body and brain weight correlations in control and PAE offspring with litter size at E14 and P0. **(A, B)** Brain weight correlations with body weight in E14 and P0 Control and PAE offspring. **(C, D)** Average body weight correlations with litter size in E14 and P0 Control and PAE litters. **(E, F)** Average brain weight correlations with litter size in E14 and P0 Control and PAE litters. (*: p < 0.05 for slope significantly non-zero).

Brain and body weights were correlated at both timepoints, although the relationship was weaker at P0 (Fig 3A and 3B). When measuring the correlation between average weights per litter and litter size, offspring in smaller litters had higher weights (Fig 3C–3F). The observation that smaller litters tend to have larger offspring has been previously reported [27]. This correlation was less strong and not statistically significant in PAE litters, suggesting that while some of the variability in the control offspring weights can be accounted for by differences in litter size, variability in the PAE group may be more driven by alcohol treatment. We observed no correlation between dam age and weights nor between dam age and litter size (S1 Fig in S1 File). Sex was also not contributing factor as we saw no different between male and female weights at E14 or P0 (S1 Fig in S1 File).

We also examined whether embryo position in the uterine horn influenced offspring weights or alcohol levels. Previous studies have shown uterine positional effects on growth, with the heaviest fetuses occupying the ovarian and cervical ends of the uterine horn, as well as positional effects on physiological functions, morphology, and behavior [27–29]. At E14, we recorded the embryo position in relation to the cervix and left versus right (S2A Fig in S1 File). Although not statistically significant, we did observe a trend of embryos closest to the cervix having higher body weights compared to those more distal (S2B Fig in S1 File), thus similar to litter size, embryo position may contribute to some of the weight variability observed in controls. This trend was not observed in PAE embryos or for Control or PAE brains (S2C Fig in S1 File). We also examined whether uterine position influenced EtOH levels in the embryo brain. Past studies of prenatal cocaine exposure in rats showed cocaine levels vary as a function of uterine position, with fetuses closest to the cervix having significantly higher levels [30, 31]. From a separate cohort, whole brains from E14 embryos were taken for gas chromatography mass spectrometry, and EtOH levels were measured 15, 30, or 60 minutes after i.p. injection of 2.9 g/kg EtOH. We found no difference in EtOH levels with respect to uterine position or comparing the left versus right uterine horn (S2D, S2E Fig in S1 File). There was, however, variation in EtOH levels across different embryos at the same timepoints. At 30 minutes, when EtOH levels are reported to peak after i.p. injection [32], there was over a four-fold difference in EtOH concentrations. Thus, while the uterine position may not be predictive of EtOH levels, not all embryos within the same litter were exposed to the same amount of alcohol.

### Variable brain weights in response to PAE correspond with differentially changes in brain transcriptomics

**Fig 4:**
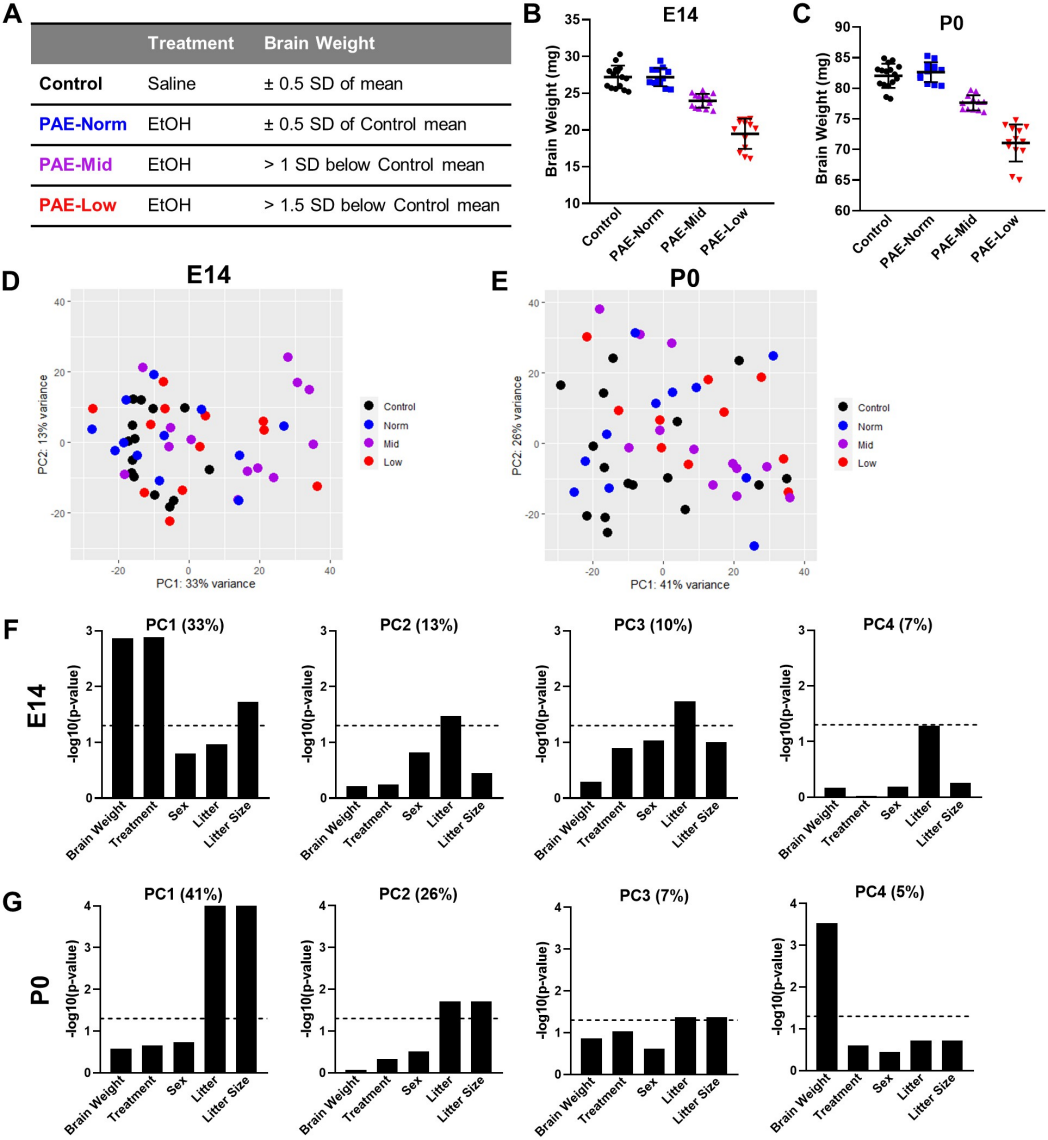
Classification of PAE mouse brains by weight relative to control shows differences in transcriptomic profiles. **(A)** Characterization of mouse embryos based on brain weight into Control (saline), PAE-Norm (normal-weight), PAE-Mid (middle-weight), and PAE-Low (low-weight) groups. **(B, C)** Brain weights by category for E14 and P0. **(D, E)** PCA plots for E14 and P0 brain transcriptomes colored by brain weight category. **(F, G)** The significance of correlation of different variables with the first four PCs in E14 and P0 brains. Values in parenthesis indicate the percent variance for each PC. The dotted line indicates P-value of 0.05.

Variation in brain and body weight suggest that some offspring within the same litter may be more vulnerable or resilient to the effects of PAE. Alternatively, as variability was also observed in the control offspring, variability in PAE weights may reflect preexisting within litter variability. To explore these hypotheses, we performed whole transcriptome expression profiling in the cortex to determine whether differential changes in brain weight after PAE result in differential gene expression. Control samples were selected based on having brain weights within 0.5 standard deviation (SD) from the average saline brain weight. PAE offspring were characterized as normal, middle, or low brain weight based on brain weights being within 0.5 SD (PAE-Norm), between 1 and 1.5 SDs (PAE-Mid), or less than 1.5 SDs (PAE-Low) of the saline mean (Fig 4A–4C). At least one normal, middle, or low weight offspring was selected from each litter. Of 84 E14 PAE offspring, 16 (19%) met criteria for PAE-Mid and 13 (15.5%) for PAE-Low. Of 80 P0 PAE offspring, 12 (15%) met criteria for PAE-Mid and 13 (16.3%) for PAE-Low at P0. Sex was roughly balanced across categories; however, more females were included for transcriptomics due to a bias towards more PAE embryos being females compared to control, particularly at E14 (Control = 45% females, PAE = 66% Females) The proportion of females was highest in the lowest weight brains (E14% Females: Control = 57, PAE-Norm = 58, PAE-Mid = 64, PAE-Low = 75). This female bias was also observed at P0 for PAE-Low (P0% Females: Control = 56, PAE-Norm = 50, PAE-Mid = 50, PAE-Low = 69). Thus, while weights did not differ by sex, the lowest weight brains for either time points were more likely to be female. Whether this is due to increased vulnerability of female offspring to have the lowest weight brains or an increased vulnerability of the most affected male embryos to premature death is unknown. Because of this bias, more females were represented in the samples used for transcriptomics due to the limited availability of male samples for PAE within our weight criteria (E14 female N/male N: Control = 8/6, PAE-Norm = 7/5, PAE-Mid = 9/5, PAE-Low = 8/3; P0 female N/male N: Control = 7/9, PAE-Norm = 6/6, PAE-Mid = 6/6, PAE-Low = 4/9).

Principal component analysis (PCA) revealed relationships of transcriptome to exposure and outcome (Fig 4D and 4E; S3 Fig in S1 File). We analyzed the association between different variables (brain weight, treatment, sex, litter, and litter size) against the first 4 PCs. At E14, we observed a significant association of brain weight, treatment, and litter size with PC1, and a significant or near significant association of litter with PCs 2, 3, and 4 (Fig 4F). At P0, there was a significant association of litter and litter size at PCs 1, 2 and 3, whereas brain weight was only significantly associated at PC4 (Fig 4G). These findings demonstrate that between litter variability in gene expression is stronger postnatally compared to embryonically, possibly due to variability introduced during birth or early postnatal care. At both E14 and P0, sex did not appear to contribute significantly to PC variance, consistent with the lack of sex effect on weights.

**Fig 5:**
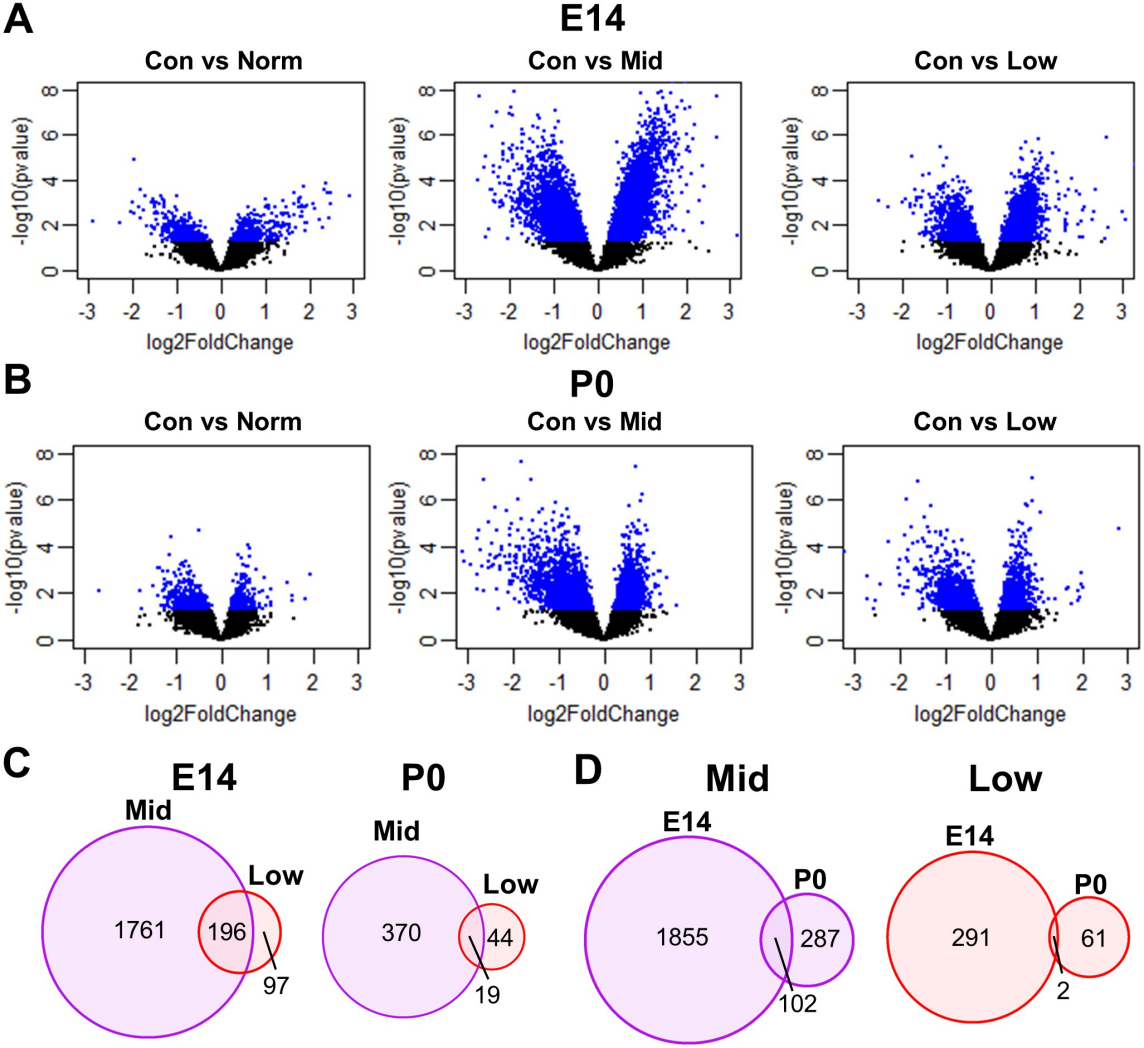
Differentially expressed genes (DEGs) and overlapping DEGs across different brain weight categories reveals the greatest differences in the PAE-Mid brains. **(A-B)** Volcano plot showing differentially gene expression between Control and PAE for E14 and P0 across the different brain weight categories. Data points in blue represents genes that were significantly different at a p-value <0.05. **(C)** Venn diagram shows the overlapping FDR significant (adjusted p-value <0.05) DEGs for Con vs Mid and Con vs Low at E14 and P0. **(D)** Venn diagram shows the overlapping FDR significant (adjusted p-value <0.05) DEGs for E14 and P0 for the Control vs Mid and Control vs Low comparisons.

Differentially expressed genes (DEGs) distinguished Control from PAE-Norm, Mid, and Low brains (Fig 5A and 5B). Remarkably, no FDR significant (adjusted p-value < 0.05) DEGs were detected for PAE-Norm at either E14 or P0 (Table 1A), suggesting these offspring were both phenotypically as well as transcriptomically minimally affected by alcohol. Unexpectedly, while we predicted that the PAE-Low offspring would show the greatest transcriptomic differences, PAE-Mid offspring showed the most significant changes. One potential reason could be that PAE-Low embryos have more transcriptomic variability, where the significant reduction in brain weight may be due to a variety of mechanisms or associated with other effects. PAE-Mid brains would thus represent a more consistent response to alcohol. When determining the coefficient of variation (CV) for PCA values in PC1, the PAE-Low brains indeed showed the highest variability at both timepoints (E14 CVs: Control = 0.63, PAE-Norm = 2.94, PAE-Mid = 1.30, PAE-Low = 13.8; P0 CVs: Control = 3.97, PAE-Norm = 8.84, PAE-Mid = 1.63, PAE-Low = 20.8). Consistent with the lesser effect of PAE on brain weight at P0 compared to E14, there were fewer DEGs detected in both the PAE-Mid and PAE-Low groups at P0 than E14. This may also be in part due to the higher transcriptomic variability observed at P0, decreasing the power to detect statistically significant DEGs. We did observe a shift in the directionality of DEG expression from E14 to P0, wherein more DEGs were upregulated at E14 and downregulated at P0. This shift suggests that PAE more transiently activates gene expression, whereas the lasting effects appear to involve downregulation of gene expression.

**Table 1:** Differentially expressed genes (DEGs) across conditions of alcohol exposure and response. Genes were considered a DEG with an adjusted p-value of less than 0.05.

	Total	Downregulated	Total
**A. Control vs PAE**			
**E14**			
PAE-Norm vs Control	0	0	0
PAE-Mid vs Control	1957	725 (37%)	1232 (63%)
PAE-Low vs Control	293	74 (25%)	219 (75%)
**P0**			
PAE-Norm vs Control	0	0	0
PAE-Mid vs Control	389	276 (71%)	113 (29%)
PAE-Low vs Control	63	37 (59%)	26 (41%)
**B. PAE vs PAE**			
**E14**			
PAE-Mid vs PAE-Norm	424	170 (40%)	254 (60%)
PAE-Low vs PAE-Norm	1	0	1 (100%)
PAE-Low vs PAE-Mid	73	15 (21%)	58 (79%)
**P0**			
PAE-Mid vs PAE-Norm	11	10 (91%)	1 (9%)
PAE-Low vs PAE-Norm	0	0	0
PAE-Low vs PAE-Mid	60	6 (10%)	54 (90%)
**C. Averaged Litters**			
**E14**			
PAE vs Control	23	5 (22%)	18 (78%)
**P0**			
PAE vs Control	1	0	1 (100%)

Further supporting the observation of within litter differences in molecular responses to PAE, within PAE comparisons of PAE-Norm, Mid, and Low brains revealed several DEGs, with comparisons against PAE-Mid brains showing the greatest changes (Table 1B). At E14, PAE-Norm versus PAE-Mid showed the greatest number of DEGs, and similar to Control versus PAE-Mid most were upregulated. At P0, fewer DEGs were detected across all comparisons. The decreased number of DEGs between PAE-Mid and PAE-Low versus PAE-Norm compared to PAE versus Control may be due to the greater transcriptomic variation of PAE-Norm brains compared to Control, as indicated by their increased variability in PC1.

We additionally averaged the gene expression profiles of all offspring within each litter (S4 Fig in S1 File). Only 23 DEGs were observed between Control and PAE litters at E14 and only 1 DEG at P0 (Table 1C). Thus, without phenotypically characterizing offspring, transcriptomic changes in mice may be masked by unaffected offspring or offspring with the more extreme, variable phenotypes. Of the DEGs detected at E14 in litter-based analyses, all were also DEGs for PAE-Mid, and 70% were DEGs for PAE-Low.

We also compared the DEGs which overlapped across brain categories and treatment groups (Fig 5C and 5D). 67% of genes altered in PAE-Low were also altered in PAE-Mid at E14, indicating shared mechanisms on brain size reduction (Table 2). In contrast, only 30% of the genes in the PAE-Low overlapped with PAE-Mid at P0, suggesting that despite initial shared mechanisms, middle and low weight brains diverge transcriptomically after alcohol exposure ceases. At both E14 and P0, the overlap of DEGs when comparing the PAE-Mid to Control versus comparing to PAE-Norm was high (84% and 82%, respectively). For PAE-Mid, 26% of genes altered at P0 were also altered at E14, these genes representing transcriptomic changes in response to alcohol that persist after cessation of PAE. For PAE-Low, only 3% of the genes altered at P0 were altered at E14. Therefore, PAE-Mid offspring are not only most affected by alcohol treatment in regard to total transcriptomic changes, several of the genes that were altered more closely to the treatment remain altered at birth, whereas gene changes in PAE-Low brains are not as persistent or are less detectable due to the increased variability.

**Table 2:** Overlapping DEGs across conditions of alcohol exposure and response. #DEGs indicates the number of DEGs for each comparison. Overlap indicates the percent overlap of Comparison 2 in Comparison 1, with the last #DEGs column indicating the number of overlapping DEGs.

Comparison 1	#DEGs	Comparison 2	#DEGs	Overlap	#DEGs
**E14**					
PAE-Mid vs Control	1957	PAE-Low vs Control	293	67%	196
PAE-Mid vs Control	1957	PAE-Norm vs PAE-Mid	424	84%	358
PAE-Mid vs Control	1957	PAE-Mid vs PAE-Low	73	59%	43
PAE-Low vs Control	293	PAE-Mid vs PAE-Low	73	2.8%	2
**P0**					
PAE-Mid vs Control	389	PAE-Low vs Control	63	30%	19
PAE-Mid vs Control	389	PAE-Norm vs PAE-Mid	11	82%	9
PAE-Mid vs Control	389	PAE-Mid vs PAE-Low	60	47%	28
PAE-Low vs Control	63	PAE-Mid vs PAE-Low	60	0%	0
**E14 vs P0**					
PAE-Mid vs Control	1957	PAE-Mid vs Control	389	26%	102
PAE-Low vs Control	293	Con vs PAE-Low	63	3%	2

### Genes and pathways affected by PAE across different brain weights

**Fig 6:**
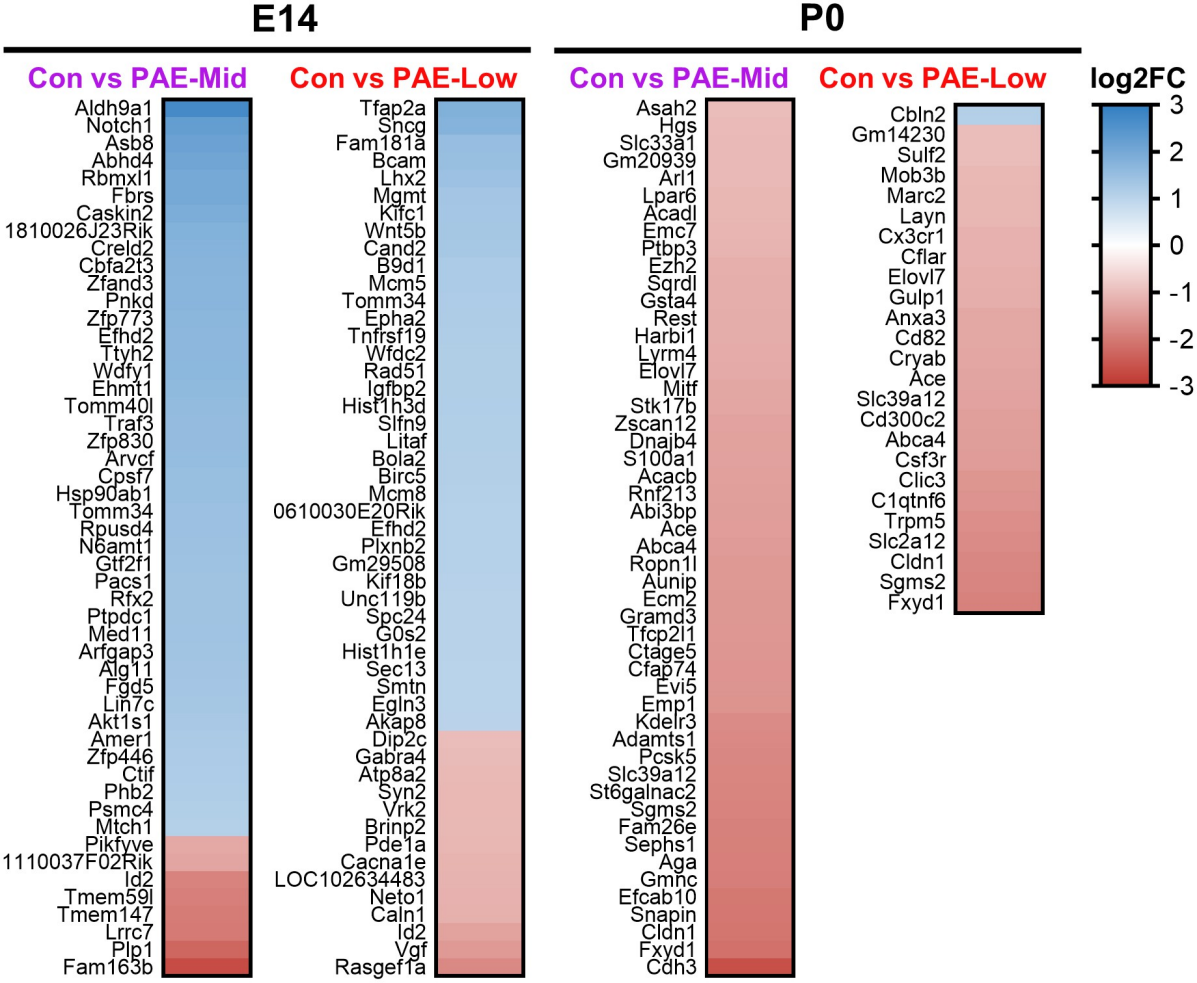
Top DEGs in E14 and P0 mid-weight and low-weight brains. Heatmap of the top 50 DEGs in E14 and P0 Control vs. PAE-Mid and Control vs PAE-Low comparisons. The top DEGs shown are those with log_2_ fold change > 1 and are ranked by adjusted p-value.

The top 50 DEGs by adjusted p-value with a log2 fold change (FC) > 1 for each Control versus PAE comparison are shown in Fig 6, with only 25 DEGs for P0 PAE-Low meeting the FC criteria. Despite 67% of all DEGs overlapping between E14 PAE-Mid and PAE-Low, of the top 50 statistically significant DEGs, only three genes overlapped (*Tomm34*, *Efhd2*, and *Id2*). These genes are all broadly associated with a variety of cellular processes, including cellular growth, differentiation, and apoptosis, and all were previously implicated in FASD studies in mouse models [33, 34]. Seven genes overlapped when comparing the top DEGs at P0 PAE-Mid and PAE-Low (*Ace*, *Fxyd1*, *Cldn1*, *Slc39a12*, *Abca4*, *Elovl7*, and *Sgms2*). None of the top DEGs at E14 were also top DEGs at P0 for either mid- or low-weight brains. Other genes implicated in previous FASD studies include *Notch1* [35–37], *Abhd4* [38], *Ehmt1* [39], *Id2* [33, 40], *Lhx2* [41], and *Ezh2* [42, 43].

To determine the biological processes altered with PAE, pathway analyses were completed using DAVID. The top ten Gene Ontology (GO) biological processes are shown in Table 3. For E14 PAE-Mid, the most significant upregulated processes include cell cycle and cell division, perhaps indicating a compensatory activation of these pathways in response to PAE. Also upregulated by PAE were apoptotic process, DNA repair, protein ubiquitination, and cellular response to DNA damage stimulus, processes likely reflecting the more intermediate response to alcohol. Cell cycle, apoptotic process, and cell division were also upregulated in E14 PAE-Low brains, however the most significant pathway upregulated was rRNA processing, with ribosome biogenesis and enzyme-directed rRNA pseudouridine synthesis also upregulated. Downregulated processes in E14 PAE-Mid brains include those related to phosphorylation as well as insulin receptor signaling, and several processes related to ion and transmembrane transport were downregulated in both PAE-Mid and PAE-Low brains. Modulation of synaptic transmission, neuron projection development, and regulation of long-term neuronal synaptic plasticity were also downregulated, demonstrating a decrease in more mature neuronal processes. Fewer biological processes were detected at P0, with no significant upregulated processes detected for PAE-Low. The top upregulated pathways in the P0 PAE-Mid were mRNA processing, RNA splicing, and mRNA splicing; and lipid metabolic process, metabolic process, and actin cytoskeleton organization were the top downregulated processes. Only two pathways were significantly downregulated in the P0 PAE-Low brains, lipid metabolic process and transmembrane transport, both of which were also downregulated in P0 PAE-Mid. Five of the eight total upregulated pathways for P0 PAE-Mid brains were also upregulated at E14 (proteasome-mediated ubiquitin-dependent protein catabolic process; RNA splicing; mRNA splicing, via spliceosome; protein ubiquitination; and mRNA processing), suggesting that many processes upregulated at P0 might be residual from E14. In contrast, only four of 26 downregulated P0 PAE-Mid processes were also altered at E14 (transmembrane transport; phosphorylation; response to insulin; and peptidyl-threonine phosphorylation), suggesting the more later effects of PAE on brain development involve the downregulation of biological processes.

**Table 3:** Gene Ontology (GO) biological processes. Top 10 biological processes altered in E14 and P0 mid-weight and low-weight brains. The top biological processes by p-value are shown with a p-value > 0.05 and at least 3 genes represented in each process. (Genes: number of DEGs included in each process).

Upregulated				Downregulated			
Pathway	GO ID	Genes	PValue	Pathway	GO ID	Genes	PValue
**E14 Control vs PAE-Mid**							
cell cycle	0007049	68	2E-07	phosphorylation	0016310	36	1E-04
cell division	0051301	47	8E-07	protein phosphorylation	0006468	37	1E-04
apoptotic process	0006915	66	2E-06	negative regulation of calcium ion-dependent exocytosis	0045955	5	1E-04
DNA repair	0006281	43	2E-05	ion transport	0006811	35	1E-04
protein ubiquitination	0016567	45	5E-05	regulation of ion transmembrane transport	0034765	13	9E-04
phosphorylation	0016310	58	5E-05	potassium ion transmembrane transport	0071805	12	1E-03
cellular response to DNA damage stimulus	0006974	51	7E-05	insulin receptor signaling pathway	0008286	9	1E-03
negative regulation of canonical Wnt signaling pathway	0090090	20	9E-05	rhythmic process	0048511	13	2E-03
tRNA processing	0008033	16	2E-04	modulation of synaptic transmission	0050804	12	2E-03
peptidyl-serine phosphorylation	0018105	23	3E-04	neuron projection development	0031175	14	2E-03
**E14 Control vs PAE-Low**							
rRNA processing	0006364	12	4E-07	regulation of ion transmembrane transport	0034765	7	4E-06
cell cycle	0007049	19	7E-05	ion transport	0006811	11	8E-06
apoptotic process	0006915	19	8E-05	regulation of neuronal synaptic plasticity	0048168	4	9E-05
cell division	0051301	14	1E-04	potassium ion transmembrane transport	0071805	5	5E-04
neural tube closure	0001843	7	4E-04	transmembrane transport	0055085	7	2E-03
chromosome segregation	0007059	7	7E-04	regulation of long-term neuronal synaptic plasticity	0048169	3	3E-03
ribosome biogenesis	0042254	7	9E-04	regulation of protein localization to plasma membrane	1903076	3	4E-03
positive regulation of transcription from RNA polymerase II promoter	0045944	25	1E-03	regulation of membrane potential	0042391	4	5E-03
enzyme-directed rRNA pseudouridine synthesis	0000455	3	1E-03	signal transduction	0007165	11	6E-03
cellular response to UV	0034644	5	3E-03	potassium ion transport	0006813	4	7E-03
**P0 Control vs PAE-Mid**							
mRNA processing	0006397	8	3E-03	lipid metabolic process	0006629	25	3E-06
RNA splicing	0008380	7	3E-03	metabolic process	0008152	9	1E-03
mRNA splicing, via spliceosome	0000398	5	1E-02	actin cytoskeleton organization	0030036	10	2E-03
response to cocaine	0042220	3	2E-02	positive regulation of inflammatory response	0050729	6	6E-03
protein modification process	0036211	3	2E-02	heart development	0007507	10	8E-03
protein ubiquitination	0016567	7	2E-02	fatty acid metabolic process	0006631	8	9E-03
proteasome-mediated ubiquitin-dependent protein catabolic process	0043161	5	2E-02	chromosome segregation	0007059	6	9E-03
protein targeting to membrane	0006612	3	3E-02	telomeric heterochromatin assembly	0031509	3	9E-03
				apoptotic process	0006915	16	9E-03
				phosphorylation	0016310	15	1E-02
**P0 Control vs PAE-Low**							
				lipid metabolic process	0006629	6	4E-03
				transmembrane transport	0055085	4	3E-02

## Discussion

In this study, we measured the effect of PAE from E7 to E13 on brain weights and the transcriptome at E14 and P0. We found significant reductions in brain and body weights at both timepoints, with variability in weights observed between and within litters. To determine whether variability in weights predicted differences in gene expression, we classified PAE offspring based on their brain weight relative to the average control weight, with normal, middle, and low weight brains represented per litter. We found no significant changes in gene expression when comparing control and PAE-Norm brains, suggesting these offspring are relatively unaffected both phenotypically and molecularly. In contrast, PAE-Mid and PAE-Low brains showed significant changes in gene expression, and as was initially surprising, the PAE-Mid brains showed the most DEGs at both timepoints. The effects on gene expression were less robust at P0 compared to E14, reflecting the lesser effect of PAE on brain weights at P0. Pathway analysis revealed an upregulation of biological processes related to both the cell cycle as well as apoptosis and downregulation of processes related to phosphorylation and transmembrane and ion signaling pathways at E14, whereas top P0 upregulated processes included mRNA processing, and top downregulated were metabolic processes.

There are a number of variables which might influence differences in PAE susceptibility in mice not measured in this study. Mice within the same litter or different litters at the same gestational age, based on vaginal plug detection at E0, are shown to vary in their developmental progression, with as much as 12 hours difference in developmental staging among littermates [44]. Differences at that scale can result in very different transcriptomic profiles both at baseline and in response to a perturbagen, and peak alcohol levels could be reached in different embryos at different times which might represent different windows of PAE vulnerability. PAE also often result in developmental delay, and this might result in a number of gene expression changes more related different developmental staging rather than alcohol teratogenesis, complicating the interpretation of findings regarding the mechanisms of alcohol versus more general downstream consequences of developmental delay. Efforts to account for variability in developmental staging of embryos both within and across litters have utilized somite number or the appearance of the maturing forelimb as a marker in mice, with a past study reporting several developmental sub-stages and littermates varying significantly in sub-stages [45]. Thus, to gain a full understanding of alcohol teratogenesis, finer resolution of developmental staging may be necessary. Additionally, weight variability in the control offspring suggests ethanol-independent effects on weight, which might be due to different developmental staging as described or other unaccounted factors. By comparing PAE groups to controls with 0.5 SD of the mean, the effects are potentially confounded. Due to so few offspring meeting the same weight criteria as PAE-Mid and -Low in the Control group, a comparison of weight-matched Control to PAE-Mid and -Low weight brains could not be performed.

Uterine effects are also caused by differing hormonal environments based on the sex of neighboring fetuses. Sex hormones are able to diffuse through the amniotic fluid between embryos, and mouse offspring surrounded by zero versus two males show differences in physiology, morphology, and behavior [29]. Only offspring selected for transcriptomics were sexed in this study, so we do not have information regarding the effect of neighboring males, however, the sex of intrauterine neighbors may also contribute to PAE variability.

Characterizing offspring phenotypically may allow for better detection of DEGs by decreasing noise caused by variability. As such, we did observe some novel or less reported gene associations with PAE. The DEG with the highest fold-change increase in E14 PAE-Mid brains was the aldehyde dehydrogenase gene *Aldh9a1*. Although primarily expressed in liver, Aldh9a1 is also expressed in the brain and is involved in the production of the neurotransmitter GABA [46]. Changes in GABA-A receptor expression and GABAergic interneuron function after PAE have been previously observed [47, 48], and we did find changes in some GABA-A receptor subunits at E14 (*Gabrr2* increased in PAE-Mid, *Gabrb1* decreased in PAE-Mid, and *Gabra4* decreased in PAE-Mid and PAE-Low). GABA is implicated in many processes of neurogenesis [49]. These results support a role for the GABAergic system in PAE-induced changes in brain development. The second most significantly upregulated gene in E14 PAE-Mid brains (as well as a DEG for E14 PAE-Low and in averaged litters) was the neurogenic locus notch homolog protein 1 (*Notch1*). The onset of neurogenesis is driven by Notch signaling [50], and Notch1 regulates the size of the cortical progenitor pool during forebrain development [51]. As premature neuronal differentiation of progenitor cells is a proposed mechanism of microcephaly, an increase in *Notch1* may contribute to this phenomenon. A number of biological processes where *Notch1* was implicated were found to be upregulated in E14 PAE-Mid brains, including regulation of somitogenesis and homeostasis of number of cells within a tissue. Notch1 expression was shown to be altered by PAE in previous studies, including the rat placenta [36], cultured radial glial cells [35], mouse and human cultured cortical slices [52], and mouse and zebrafish heart tissue [37, 53], however with differing directionality of Notch1 expression changes in these studies. The gene with the highest fold-change in PAE-Low brains was transcription factor Ap-2 alpha, *Tfap2a*, which was not a DEG in the PAE-Mid brains. Tfap2a is important for the development of several tissues, including the nervous system where it appears to regulate the proliferation, survival, and specification of different neuronal cell types [54]. Studies in cell-derived embryoid bodies treated with ethanol *in vitro* similarly showed an increase in *Tfap2a* expression [55].

At P0, the DEGs with the largest fold change in the PAE-Mid brains were *Cdh3* (P-Cadherin) and *Fxyd1* (FXYD domain containing ion transport regulator 1), the later also being the most significantly altered gene in P0 PAE-Low brains. Cadherins are a large family of proteins involved in cell-to-cell adhesion, are important for cortical structure during development, and have been shown to be dysregulated in past PAE studies [56]. While E- and N-cadherins have been most studied in general and in the context of PAE, less is known about the developmental role P-cadherin, which is highly expressed in the placenta of mice, glioblastoma cells, and neocortex [57–59]. Fxdy1 regulates Na^+^/K^+^-ATPase activity in brain and cardiac tissue, where in the former it modulates neural excitability [60]. In primary cerebral cultures, *Fxyd1* was downregulated after cotreatment with ethanol and nicotine where it was implicated in alterations in calcium homeostasis [61]. Several biological processes related to ion transmembrane transport where downregulated in E14 PAE-Mid and PAE-Low brains, and the downregulation of *Fxyd1* at P0 may be a related to those earlier effects.

Pathway analysis reveal several biological processes altered by PAE. In this study, cell cycle was among the top upregulated biological process in both E14 PAE-Mid and PAE-Low brains. The upregulation of cell cycle may appear counterintuitive given the decrease in brain weight, however, partial recovery of brain weights at P0 suggests compensatory mechanisms may be upregulated in response to PAE. More commonly associated with the effects of PAE, apoptotic process was a top upregulated pathway at E14. At P0, the top upregulated pathways were related to mRNA processing and splicing, which were also upregulated at E14, suggesting a key role of RNA processing in both the short and longer-term effects of PAE. RNA splicing is considered a signature in the cortex after PAE implicated with RNA-sequencing analysis [62, 63]. Metabolic processes, including lipid and fatty acid metabolism, were significantly downregulated at P0, a finding also previously reported in past PAE studies and thought to regulate later behavioral problems associated with FASD [64]. Previous imaging studies in adolescents and young adults with FASD revealed a permanent alteration in brain metabolism in several regions, including the cortex, after PAE [65], suggesting that changes in metabolic processes may be a long-term effect of the exposure as we also observed. While E14 represents the more intermediate effects of PAE (24 hours after the last exposure) and P0 the more long-term, both timepoints representing a snapshot of a particular time on the developmental trajectory. Thus, transcriptomics performed closer to the exposure (i.e., 4 hours) or longer afterward (i.e., adulthood) could better represent acute and long-term effects.

Overall, this study identified potential molecular pathways by which PAE may alter brain growth. Importantly, these molecular findings were with respect to phenotypic characterization of offspring after PAE by brain weight. Within litter variability in response to PAE and other developmental perturbations, even in inbred strains, is long known and often ignored, and this study highlights that variability at the molecular level. Understanding within litter differences in rodents, the primary preclinical models for FASD, can enhance insights into mechanisms of PAE. These within litter differences should be considered in the experimental design of PAE rodent studies, where variables such as litter size, the number of males and females, and uterine position can be accounted for. While these within litter differences are observed in developmental studies, within strain differences are also widely observed in the literature regarding behavioral responses to drugs, including alcohol [66, 67], and it is reasonable to believe that these differences are at least in part developmental in nature, perhaps starting *in utero*. We hope these findings highlight both the complications and benefits of studying FASD in rodent models where large litters offer the opportunity to evaluate the reasons why animals with the same genotype and similar exposures have different teratogenic responses to alcohol and allowing for the identification of molecular mechanisms of FASD.

## Supporting information

S1 File Supporting information figures. (DOCX)

S2 File Differential gene expression results. (XLSX)
