# Supplementary material for: Differential effects of prenatal alcohol exposure on brain growth reveals early upregulation of cell cycle and apoptosis and delayed downregulation of metabolism in affected offspring
Source: PLoS One. 2024 Nov 27;19(11):e0311683. doi: 10.1371/journal.pone.0311683 (PMC11602053; doi:10.1371/journal.pone.0311683)
Supplement: S1 File — (DOCX) [file pone.0311683.s001.docx]

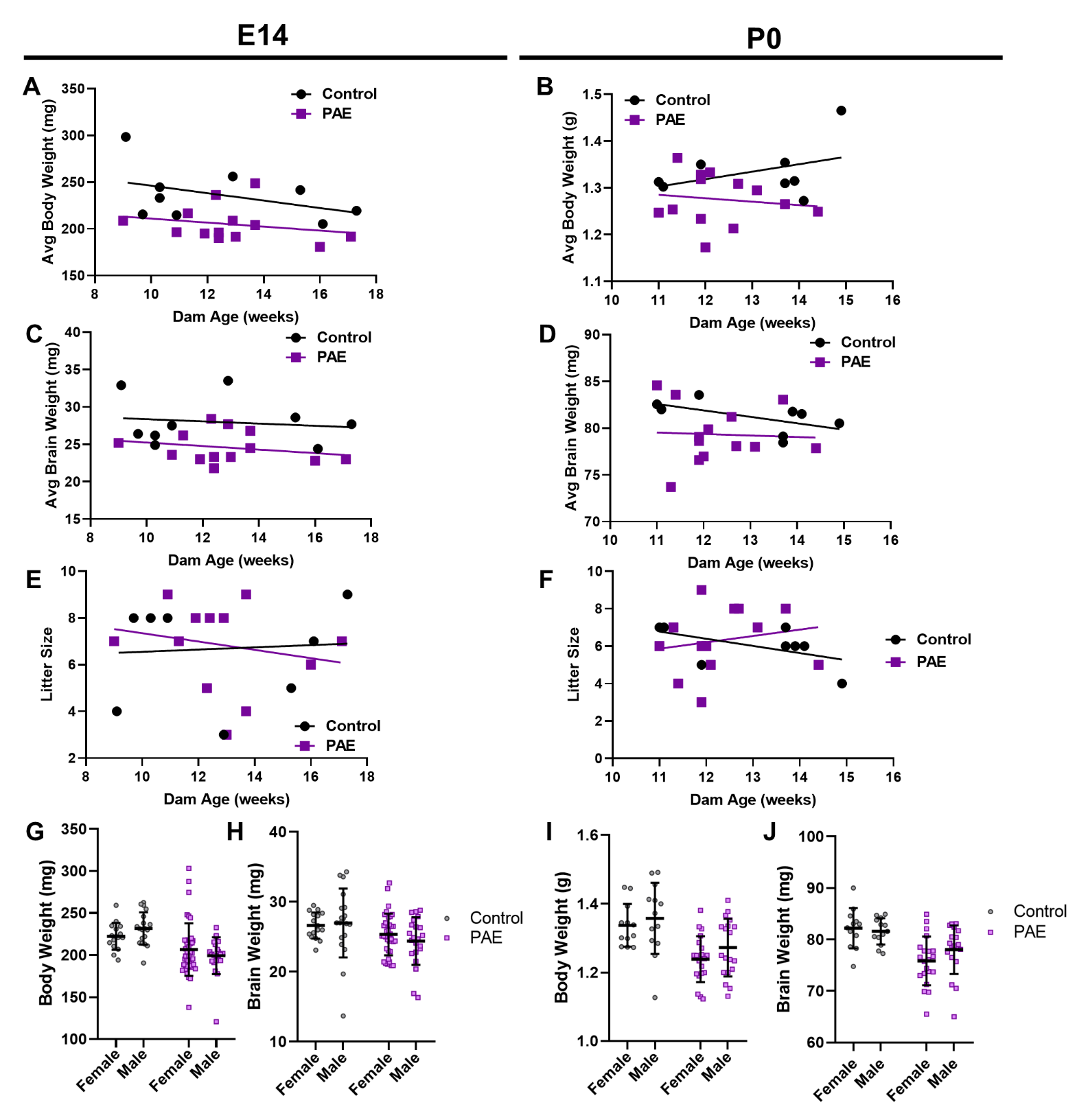


**S1 Fig. Associations of body and brain weights in Control and PAE offspring with dam age and sex at E14 and P0. (A, B)** Brain weight correlations with dam age at E14 and P0 in Control and PAE offspring. **(C, D)** Body weight correlations with dam age at E14 and P0 in Control and PAE offspring. **(E, F)** Litter size correlations with dam age in E14 and P0 Control and PAE offspring. **(G, H)** Body and brain weights of E14 Control and PAE offspring by sex. **(I, J)** Body and brain weights of P0 Control and PAE offspring by sex.

**
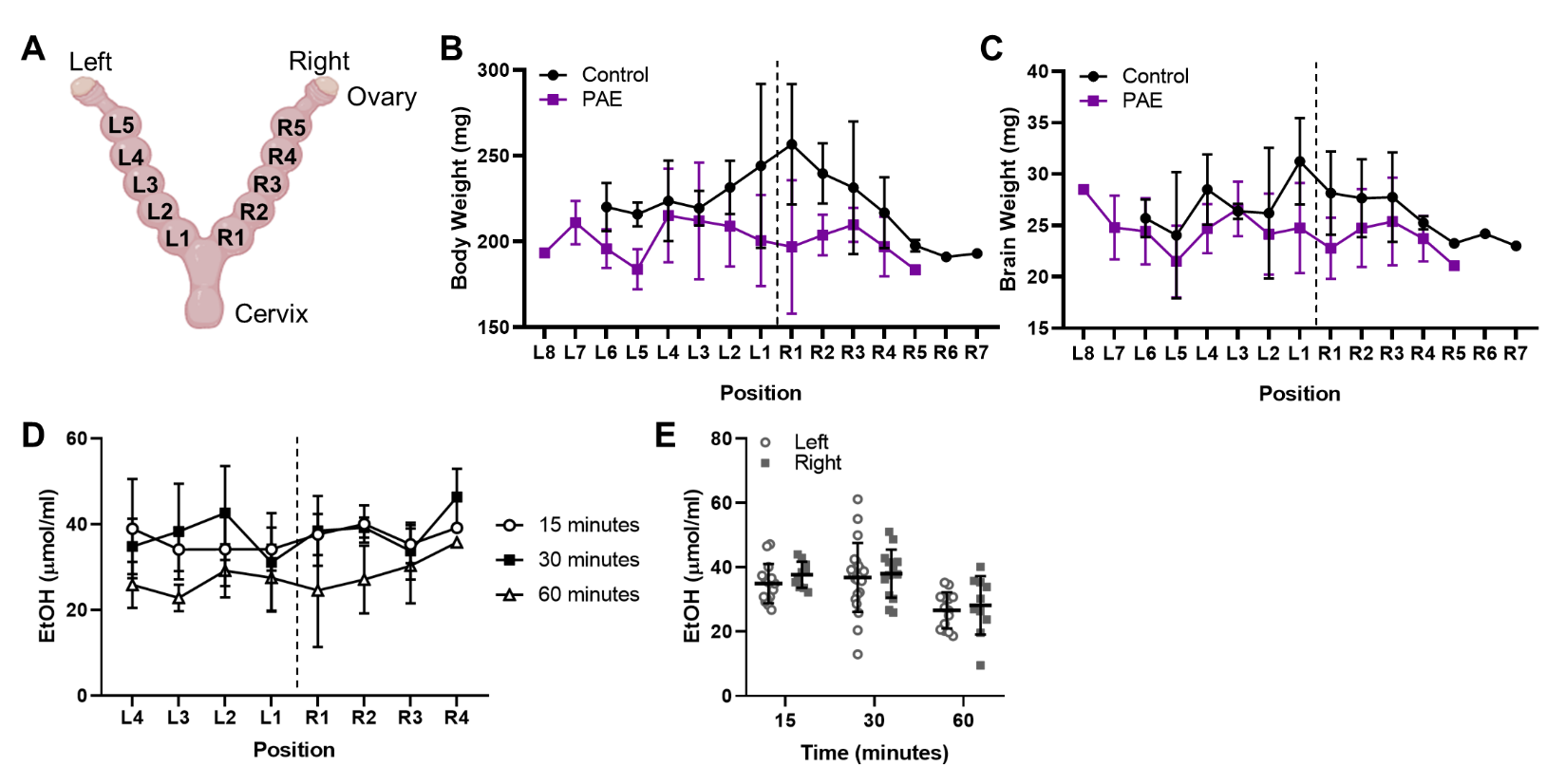
**

**S2 Figure. E14 brain weights, body weights, and EtOH brain levels by uterine position. (A)** Schematic of the mouse uterine horn, noting the identification of embryos based on uterine position. **(B, C)** Brain and body weights by uterine position in Control and PAE offspring. **(D)** EtOH concentrations, determined by GC/MS, in E14 embryo brains 15, 30, and 60 minutes after treatment with 2.9 g/kg EtOH by i.p. injection, by uterine position. **(E)** EtOH concentrations in E14 embryo brains by left or right uterine horn placement. Graphics were obtained from Biorender.com.

**
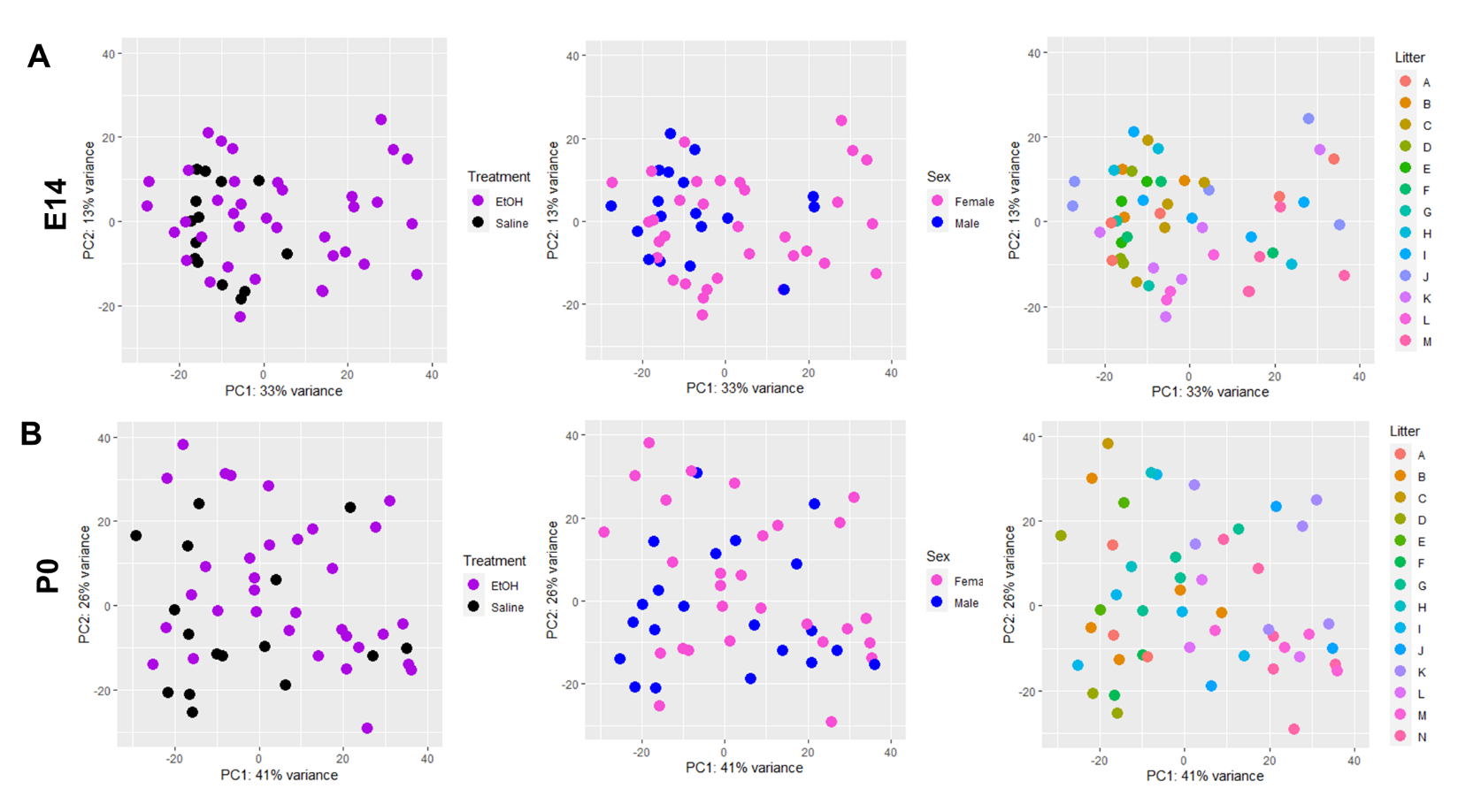
**

**S3 Figure. PCA plots. (A, B)** PCA plots of E14 and P0 brain transcriptomes for colored by treatment, sex, and litter.


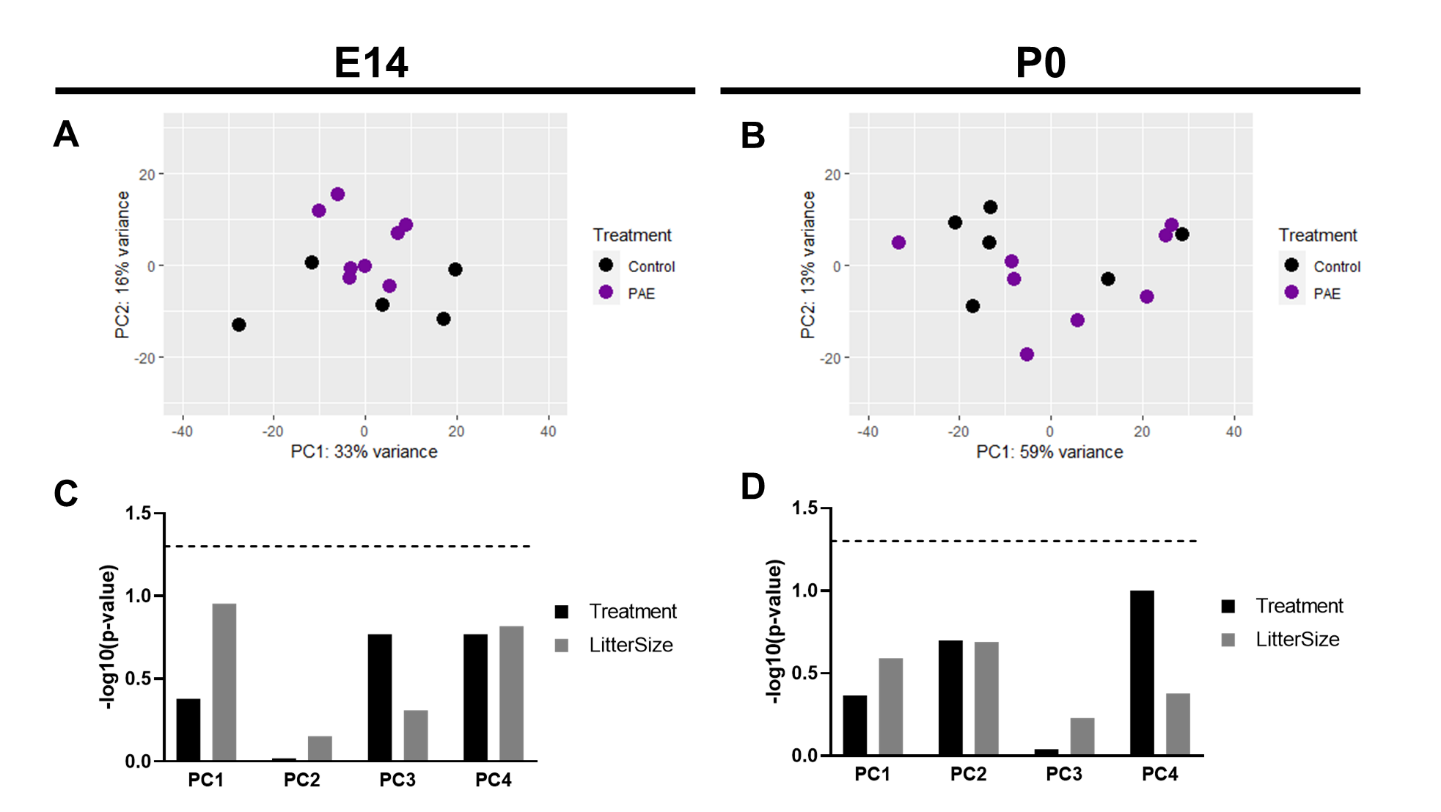


**S4 Figure. Averaged transcriptomic profiles across litters. (A, B)** PCA plots for E14 and P0 averaged transcriptomes per litter. **(C, D)** The significance of correlation of different variables with the first four PCs in E14 and P0 averaged transcriptomes.
